# Changing the Narrative: Structural Barriers and Racial and Ethnic Inequities in COVID-19 Vaccination

**DOI:** 10.3390/ijerph18189904

**Published:** 2021-09-20

**Authors:** Anuli Njoku, Marcelin Joseph, Rochelle Felix

**Affiliations:** 1 Department of Public Health, College of Health and Human Services, Southern Connecticut State University, 144 Farnham Avenue, New Haven, CT 06515, USA; josephm9@southernct.edu (M.J.); felixr4@southernct.edu (R.F.); 2 Greater Bridgeport Area Prevention Program, 1470 Barnum Avenue, Suite 301, Bridgeport, CT 06610, USA

**Keywords:** COVID-19/Coronavirus, race/ethnicity, health disparities, racism, structural racism, blacks/African Americans, Hispanic/Latino, social determinants of health

## Abstract

The COVID-19 pandemic has disproportionately affected racial and ethnic minority groups in the United States. Although a promising solution of the COVID-19 vaccination offers hope, disparities in access again threaten the health of these communities. Various explanations have arisen for the cause of disparate vaccination rates among racial and ethnic minorities, including discussion of vaccine hesitancy. Conversely, the role of vaccine accessibility rooted in structural racism as a driver in these disparities should be further explored. This paper discusses the impact of structural barriers on racial and ethnic disparities in COVID-19 vaccine uptake. We also recommend public health, health system, and community-engaged approaches to reduce racial disparities in COVID-19 disease and mortality.

## 1. Introduction

Coronavirus disease 2019 (COVID-19), the pandemic of respiratory disease spreading from person-to-person, presents an international public health emergency [1,2,3,4]. As of 15 September 2021, over 226.7 million cases of COVID-19 and over 4.6 million COVID-19-related deaths have been reported worldwide. The United States (U.S.) has the highest number of COVID-19 deaths, at over 682,000 people, as well as over 42.2 million cases and over 32.1 million recovered patients [5]. Currently, several variants of the virus that cause COVID-19 are circulating globally and within the United States. These variants tend to spread more quickly and easily than other variants, prompting concerns of increased hospitalizations, strain on healthcare resources, and possible deaths [6].

People at increased risk of getting COVID-19 include older adults, people with certain underlying medical conditions such as cancer, heart conditions, HIV infection, chronic kidney disease, liver disease, chronic lung diseases, dementia, or other neurological conditions, weakened immune system, Down syndrome, overweight and obesity, smoking, diabetes mellitus, pregnancy, solid organ or blood stem cell transplant, sickle cell disease or thalassemia, stroke or cerebrovascular disease, and substance use disorders [7]. In addition, U.S. data indicates that racial and ethnic minority groups are bearing a disproportionate burden of COVID-19-associated outcomes [8]. Black or African Americans, Hispanic or Latino persons, and American Indian or Alaska Native, Non-Hispanic persons are more likely to become sick with, be hospitalized for, and die from COVID-19, when compared to non-Hispanic Whites [8]. Guidelines to prevent the spread of COVID-19, including washing one’s hands often, maintaining social distancing, avoiding close contact with people who are sick, wearing a mask in public settings, and when around others who do not live in one’s household, avoiding crowds and poorly ventilated spaces, and getting a COVID-19 vaccine when it is available [7]. The COVID-19 vaccine has emerged as one of the methods to prevent COVID-19 infection, with evolving consensus that even when vaccination does not prevent infection, it substantially lowers the likelihood of severe illness and death in the event of infection [9]. Moreover, glaring racial and ethnic disparities exist in COVID-19 vaccination [10,11,12]. Across the United States, 75.88% (over 195 million people) of adults have received at least one dose of the COVID-19 vaccine [13]. Among people who have received at least one dose of the vaccine and for which data on race and ethnicity are available, 60.3% were Non-Hispanic White, 17.4% Hispanic/Latino, and 10.4% were Black, with data revealing continually lower COVID-19 vaccination rates among Black and Hispanic persons when compared to their White counterparts [12,13]. In most states, Black and Hispanic people are receiving smaller shares of COVID-19 vaccinations related to their shares of cases, total deaths, and the total population [12].

Even with recent increases in COVID-19 vaccination rates across racial and ethnic groups, gaps in COVID-19 vaccination rates still continue for Black and Hispanic people [12]. Moreover, racial and ethnic disparities in vaccine uptake persists, even as supply increases significantly [14]. Although the Centers for Disease Control and Prevention (CDC) has provided national-level data, state-level data on the racial and ethnic composition of those receiving the COVID-19 vaccine is not currently reported [12]. Incomplete data have resulted in missing race or ethnicity information and highlights the need for more complete reporting of race and ethnicity data at the jurisdictional and provider levels for immediate response to possible disparities in COVID-19 vaccine administration [15]. Therefore, it is imperative to explore strategies to achieve equity in access to and administration of COVID-19 vaccination among racial and ethnic minority groups who have been disproportionately affected by COVID-19 [14,16,17]. The purpose of this article is to detail the impact of structural barriers on racial and ethnic disparities in COVID-19 vaccine uptake, with a particular focus on Black and Hispanic/Latino populations. We also outline approaches to achieve efficiency and equity in the COVID-19 vaccination campaign to help reduce to reduce racial disparities in COVID-19 disease and mortality.

## 2. Racism and Health

Although the COVID-19 vaccine has emerged as a method to prevent COVID-19 infection, disparities in access to and confidence in the COVID-19 vaccine have jeopardized the health of Black communities. The terms vaccine hesitancy and medical mistrust have shaped the discourse around racial and ethnic disparities in COVID-19 vaccine uptake [18]. Vaccine hesitancy refers to “delay in acceptance or refusal of vaccination despite the availability of vaccination services” [19] (p. 4161). Mistrust of medicine and science is steeped in an extensive history of unethical procedures and research on Black communities in the United States [20]. Although historical events like the Tuskegee Syphilis Study are cited to describe why Black Americans are less certain than White Americans to obtain the COVID-19 vaccine, the Tuskegee Study can be seen as a scapegoat for contemporary racism and barriers to healthcare [21]. Moreover, researchers and practitioners in recent decades have pinpointed racism as a continuing population health crisis and a root cause of disease within the U.S. and worldwide. Studies have comprehensively established the multi-layered relationships between racism and health outcomes among racial minorities and immigrants [22,23,24,25]. Researchers have argued that racism operates at many levels, ranging from interpersonal to structural [26,27]. Structural racism is considered the most fundamental of the levels of racism and the one in most need of being addressed to achieve meaningful change, due to its multifaceted, systemic, and multi-generational effects on health outcomes among communities of color [27]. Structural racism can manifest itself as racial discrimination in policymaking and enforcement of regulation, practices, and laws, which systematically result in exclusion from or differential access to societal resources and opportunities based on race [28]. A systematic review of methods to define and measure exposure to structural racism revealed a range of measures across domains of socioeconomic status, social institutions, housing and residential patterns, workplace environments, political participation, criminal justice, and immigration [29]. Structural racism affects health through its past and current effects on quality of, and equal access to, critical social, and environmental determinants of health. For example, the practice of redlining inhibited communities of color from acquiring residential mortgages and accordingly, access to public transportation, supermarkets, and healthcare, contributing greatly to the proliferation of residential segregation in the United States [30,31,32,33]. Accordingly, in communities plagued by segregation, Black persons and other racial and ethnic minority groups in the United States are more likely to reside in neighborhoods with increased levels of poverty, have lesser access to credit, employment, housing, transportation, educational, nutritional, and healthcare resources, and live in health-limiting environments, compared to the White population [32,34,35,36,37,38]. Structural racism has been associated with reduced healthcare quality and discriminatory and excessive incarceration practices that disadvantage Black people [30,39]. Systemic racism also obstructs access to vital health care services, such as access to health insurance, a health care provider, convenient locations to receive the COVID-19 vaccine, and credible information about vaccines among racial and ethnic minorities [40]. Moreover, historical injustices perpetuate contemporary structural barriers, such as cost, convenience, language barriers, lack of transportation, and poor internet access that contribute to barriers to COVID-19 vaccination for Black and Hispanic/Latino people [41,42,43]. 

In the United States, recent increases in immigration enforcement, including unprecedented levels of deportation, has resulted in adverse impacts on health and well-being, including reduced access to vital health resources [44]. Furthermore, there is a large percentage of documented immigrants that identify as Hispanic/Latino who are also disproportionately represented in COVID-19-related sickness, hospitalizations, and deaths, which can be partly attributed to U.S. immigration policies in place throughout the first year of the pandemic in the United States, prompting people without lawful documentation to excessively avoid accessing COVID-19 testing and other community resources [45]. Overall, social determinants of health, such as health care access and quality and neighborhood and built environment, contribute to extensive health disparities and inequities [46]. Moreover, policies that systematically exclude access and fuel racial inequities in health outcomes include racial bias in various aspects of the criminal legal system, such as policing and incarceration [47,48], regulations and laws that perpetuate inequalities in exposure among racial and ethnic minority communities to environmental pollutants in housing, working conditions, air, water, and soil [49,50,51,52,53], and unequal healthcare exhibited in the form of the eugenics movement during the early 20th century in the United States [54]. 

In the health care setting, structural racism manifests to perpetuate racial and ethnic disparities, seen in how often racial and ethnic minorities are not afforded life-saving care after cardiac arrest [55]. Furthermore, Black and Hispanic/Latino patients have significantly higher in-hospital mortality rates compared to White patients, even after controlling for socioeconomic status [56]. Despite a rise in health insurance coverage since passage of the 2010 Affordable Care Act, American Indian/Alaska Native persons, Hispanic/Latino persons, and Black persons are more likely to lack health insurance compared to their White and Asian counterparts [57]. Even as Medicaid expansion authorized by the Affordable Care is associated with decreased rates of being uninsured, at least 12 states, mostly in the Southern United States and some with higher proportions of historically marginalized groups, have not granted this additional coverage [58]. Overall, these historical and contemporary patterns and practices reinforce discriminatory resource distributions and illustrate how structural racism continues to proliferate disparities across these interconnected systems that ultimately influence health.

## 3. Racism and COVID-19 

The COVID-19 pandemic has revealed the disparate health outcomes of structural racism among Black, Hispanic/Latino, and American Indian/Alaska Native American populations. These groups compose a higher proportion of frontline workers who are more likely to be exposed to the virus through work, less likely to have access to health insurance and high-quality health care to uphold their health, and have a higher occurrence of underlying medical conditions that make them susceptible to COVID-19-related sickness, hospitalization, and death [58,59,60]. To illustrate the effect of structural racism on COVID-19 outcomes, racial disparities in socioeconomic outcomes could result in racially patterned differences in access to health-sustaining resources, such as adequate shelter, nutritious food, healthcare, masks, and sanitizers, along with racially disparate environmental and occupational risks [29,59]. For instance, Black adults are less likely to own their homes compared to White adults, regardless of education or income. In another example, Native Americans are 19 times more likely to lack complete plumbing and Black and Hispanic/Latino households are nearly twice as likely to lack complete plumbing when compared to White households, which obstructs ability to wash one’s hands and prevent the spread of COVID-19 [61]. People without lawful immigration status in the United States identify overwhelmingly as Hispanic/Latino and are disproportionately represented in high-exposure frontline industries including construction, farming, groundskeeping, food production and delivery, cleaning and sanitation services, and waste management, putting them at increased risk for COVID-19 exposure [62,63]. Housing insecurity, concentrated poverty, overcrowding, environmental hazards, and reduced access to health providers continue to afflict historically marginalized communities, and have contributed to increased risk of COVID-19 sickness illness [58]. 

Moreover, COVID-19 reveals the lingering medical mistrust and mistreatment in the Black community. Racism and COVID-19 present a double pandemic for Black communities, contributing to poorer COVID-19 outcomes [64]. Racial bias has been seen in COVID-19 testing, illustrated by the several well-known cases of denied access to COVID-19 testing and treatment among Black Americans [65,66,67,68]. Studies have shown that despite being at greater risk of exposure to COVID-19 and needing more intensive care once testing positive for COVID-19, Black patients do not have noticeably higher testing rates and face greater barriers obstacles to health care [69,70]. Hence, inequalities in access to medical testing, diagnosis, and management increase the susceptibility to COVID-19 among U.S. Black and Hispanic communities [71]. Moreover, the recognized risk factors for COVID-19 should be examined within the context of the unfavorable social determinants of health that place Black communities at greater risk for sickness and death. Such factors involve, but are not limited to, income, education level, occupation, reduced access to healthy food, housing density, and crowding conditions [54].

Furthermore, while the latest reports show that enthusiasm for the COVID-19 vaccine continues to grow and that a decreasing number of Black adults want to “wait and see” before they get vaccinated, the share of Black adults waiting to get vaccinated is still higher than those of White adults. Additionally, large shares of Hispanic adults and those with lower incomes are uncertain about if they are currently eligible to obtain the vaccine in their state [10]. There are also reports that vaccine rollout in minority communities is inhibited by geographic areas that have scarce access to a nearby pharmacy and where pharmacy services are limited or challenging to obtain [72,73]. The troubling statistics on who is getting the COVID-19 vaccine, and who is not, should change the narrative from hesitancy within communities of color and toward the structural barriers that breed that mistrust and influence vaccine rollout. 

## 4. Structural Barriers and COVID-19 Vaccine Access

Structural barriers are systemic factors that may hinder the ability of an individual to access vaccination [74]. There are various structural barriers to COVID-19 vaccination among Black and Hispanic/Latino people. Table 1 provides a summary of common structural barriers to vaccination services.

### 4.1. Convenience

In some situations, hesitancy is assumed to be the reason for poor uptake. However, closer examination often reveals the larger importance of factors, such as complacency, confidence, convenience, accessibility, availability, and quality of healthcare services [19,80]. Practical issues that affect vaccine hesitancy include convenience, which can be an important consideration when physical availability, affordability and ability to pay, geographical reach, ability to understand (health literacy and language), and the appeal of immunization amenities affect the decision to be vaccinated [19]. Furthermore, while vaccine hesitancy is often cited as a driver of racial and ethnic disparities in vaccination rates, it does not fully explain the variation in vaccine coverage for Black and Hispanic populations [18,43]. Moreover, this approach puts the burden on marginalized communities to increase vaccination without addressing the primary barriers to COVID-19 vaccination, including access to vaccines. Therefore, it is important to stop using vaccine hesitancy as an excuse for structural racism and acknowledge its role in keeping crucial healthcare services beyond the reach of communities of color [40]. This includes access to health insurance, a healthcare provider, credible information about vaccines, and convenient access to vaccines [40]. Other challenges associated with Black and Hispanic populations obtaining a vaccine include lack of a centralized system for residents to register for and schedule vaccination appointments, complexity of the vaccine scheduling system related to internet access, comfort with technology, having to navigate multiple online platforms, distance to travel, and not having enough information about when and where to receive a vaccine [43]. Additionally, access to COVID-19 vaccine sites may be dependent on where a person lives. Data show that COVID-19 vaccine locations tend to be disproportionately clustered in more affluent zip codes with lower minority populations and farther than where Black and Hispanic residents live [43,81]. Access issues, such as lack of prioritization of vulnerable groups and limited vaccination clinic availability, lead to greater demand, lengthier wait times for vaccination, and greater travel time to vaccine sites [16]. Overall, these convenience factors likely contribute to decreased vaccination rates in Black and Hispanic populations.

### 4.2. Immigrant Status

The federal government has allotted resources to make the COVID-19 vaccine available at no cost for people who are uninsured regardless of immigrant status, but some people still may fear that they will face out-of-pocket costs for the vaccine [82]. Non-citizen immigrants are more likely to be uninsured compared to U.S. citizens and are more likely than their counterparts to be concerned about potential costs related to obtaining the vaccination [76]. Other access-related barriers to COVID-19 vaccination among immigrants who identify as Hispanic or Latino/a include not knowing if one is eligible to receive the vaccine, worrying that obtaining the vaccination may have negative immigration-related consequences such as immigration enforcement arrests, or being asked to show proof of legal residency when that is not a requirement to obtain the COVID-19 vaccine [42,77,82]. Providers and vaccine sites collect and share information from individuals to monitor uptake, determine appropriate timing for a second dose, and assess vaccine effectiveness and safety [14]. However, there can be fear and distrust among immigrant communities about providing information without knowing how it will be used [76,81]. Other potential challenges to COVID-19 vaccination among immigrants include lack of flexibility in work and childcare responsibilities, limited transportation options, lack of access to computers, and literacy and language challenges [63,76]. Therefore, these are key issues to consider when trying to ensure equitable access to COVID-19 vaccines.

### 4.3. Language

Encountering a condition like COVID-19 is one matter, but not being able to understand the severity of the virus because of language barriers is another. Language is another barrier to COVID-19 vaccination and is a growing concern for people who do not speak or understand English. One issue for native Spanish-speakers in the U.S. has been websites with inaccurate Spanish translations, which can cause confusion and squander valuable time as appointment slots fill up [41]. Other language-related issues that hinder vaccine access include websites neglecting to mention that the vaccine is free, limited language translation help at vaccination clinics, limitations of translation software used at many state and county health department websites, and varying translation help on state vaccine-finder websites, with some states having no language help on their websites [75]. Poor translation can result in non-English speakers experiencing fear, vulnerability and skepticism around COVID-19 and an inability to express their concerns or needs [63]. This illustrates the importance of people understanding the information they receive in order to adopt a health behavior and receiving the information in a language that is meaningful to them. Therefore, it is very vital to develop a trustworthy relationship with people in order to have them take the vaccine and to communicate to people in the language they speak and understand, in order to help promote their uptake of the COVID-19 vaccine. 

### 4.4. Transportation

Transportation is often a significant barrier to care. In the United States, although 80% of Americans live in urban areas, 45% of households do not have access to transportation [83]. This has caused a barrier to travel to COVID-19 vaccination sites. There are COVID-19 vaccination sites that are a 35-min drive each way or at least an hour-long bus ride from one’s residence, posing a barrier to get to COVID-19 vaccine sites [84]. Vaccination sites are not always near public transit and not everyone has access to a car. A survey analysis revealed that in 2017, Black households were the least likely out of any racial or ethnic group in the U.S to own a car, followed by Native American households [41]. If an appointment becomes available the same day that individuals have work or childcare commitments, this is an added complication. Moreover, taking public transportation poses a possible COVID-19 risk, and ride-sharing can be costly [41]. Additionally, many low-income older adults who are most at risk for COVID-19 cannot make it to a vaccination site [84]. Many individuals who are homebound also face the challenge of transportation to a COVID-19 vaccination site [84]. Other obstacles include lack of accommodations for older adults and people with disabilities [43]. Without an effective plan in place, this will continue to increase health inequities, which will cause more health disparities surrounding COVID-19 vaccination.

### 4.5. Computer and Internet Access

Online vaccine appointment scheduling systems can reveal the deep digital divide between those who may lack access to or feel uncomfortable using the internet and those who can effectively maneuver the internet [18]. Due to the COVID-19 pandemic, many eligible participants have been encouraged to register online to get vaccinated [85]. This process requires that the user have access to a rapid digital connection. However, not everyone has access to an adequate internet connection. Navigating a complex vaccine scheduling system can be a considerable barrier for people without access to the internet or a computer or those who are not comfortable with technology. The ability to schedule vaccination appointments favors those with the flexibility and time to travel long distances or take off from work [43]. Access to a traditional computer and home broadband also differs by race and ethnicity [41]. Data have shown that Black and Hispanic adults in the United States continue to be less likely than non-Hispanic White or Asian adults to report owning a traditional computer or having high-speed internet at home [77]. Moreover, these barriers may disproportionately impact Black and Hispanic Americans, who compose a disproportionate number of essential workers who may not have the time and flexibility to travel to vaccination sites or take time off work [43]. Access issues might continue in hospital systems. Environmental services members, such as janitorial staff, may not have access to email and therefore may miss being reached with vaccine registration information, illustrating structural racism [78]. 

### 4.6. Lack of Trusted Points of Access

To address racial and ethnic disparities inequities in COVID-19 vaccination, adversely affected communities need to have trusted points of access. Vaccination programs need to meet people where they are and bring the vaccines to them [86]. The lack of prioritization of disproportionately affected groups has led to the limited vaccination clinic availability in Black and Hispanic neighborhoods [16]. For instance, the initial phases of vaccine distribution have been primarily limited to chain pharmacies and large health-care systems, which are significantly less prevalent in Black communities [79]. Moreover, racial, and ethnic minority patients are more likely to receive care in under-resourced healthcare systems [58]. There is a need for trusted points of access to the COVID-19 vaccine in Black communities, and in places such as schools, community centers, faith-based organizations, and mobile vaccination units organized by trustworthy and credible community-based organizations. These vaccination appointments at sites in Black communities should also be prioritized for residents in that community to avoid instances where ease of virtual signup may attract people from wealthier neighborhoods who have more reliable internet access and fewer access barriers [79,87].

## 5. Discussion

Examination of the barriers that contribute to COVID-19 vaccination-related health disparities among Black and Hispanic/Latino communities calls for public health, health system, and community-engaged approaches to promote equitable access to and confidence in the COVID-19 vaccine [19]. These types of barriers could be addressed by making changes to the structure and funding mechanisms of the healthcare system and industries that supply the healthcare system [74]. We propose the strategies below to address racial and ethnic inequities in access to COVID-19 vaccination, with the view that these solutions may be overlapping and serve to address several barriers simultaneously.

### 5.1. Tailor Vaccine Messaging to Communities Most at Risk

Racial and ethnic disparities in adherence to COVID-19 recommendations may be influenced by health literacy, mistrust of public health messages, and mistrust of the healthcare system, with African Americans having greater levels of mistrust [88]. To reduce racial disparities in COVID-19 disease and mortality, messages must be tailored towards and trickle down to communities most at risk. To do so, we must acknowledge that all communities are not the same. Information can be interpreted differently from one community to the next. The main universal message used when trying to encourage uptake of the COVID-19 vaccination is that the vaccine is very effective, protects one from illness, and provides an opportunity to get back to everyday life [89]. Not everyone may be responsive to this example of messaging. One study found that among White, Black, and Hispanic adults who are tentative about the vaccine, Black and Hispanic adults have a greater concern that they might experience severe side effects from the vaccine [89]. There have been cases at mass vaccination events where people in attendance who have allergy issues may not have known what version of the vaccination they received [90]. This suggests an opportunity for patients and vaccine providers to discuss credible information about possible allergic reactions to the COVID-19 vaccine along with expectations of those receiving the vaccine. 

Multiple-dose offerings could pose challenges for those who find a single dose more convenient. Moreover, rare complications or immune responses may influence the tendency to hesitate from taking medications [90]. For example, having an allergic reaction to the drug is one reason people use as a hesitation. Furthermore, many members of racial and ethnic minority communities cautiously wait to observe for themselves whether or not the vaccines are safe. They do so by speaking with family members, watching loved ones who have received the vaccine to observe any side effects, evaluating information from local leaders and trusted sources, and speaking with health care professionals [18]. It is important that these matters be considered when developing messaging in order to avoid such hesitancies. There should be messaging around what to do if one feels that they are experiencing side effects and available resources in the case of severe side effects. 

### 5.2. Carefully Consider Health Literacy and Language 

Health literacy is also an important consideration when developing messaging. Being able to obtain, process, and understand information to make decisions about one’s health can help people of color feel safe and renew their trust toward health care [91]. Increasing individuals’ health literacy through education and communication programs has been associated with acceptance of the COVID-19 vaccine [92]. There is also a crucial need for culturally responsive, targeted, and accessible public health messaging to help tackle racial disparities in COVID-19 risk [93]. This way of messaging could influence what people feel and think about the COVID-19 [94], and help improve the rate of COVID-19 vaccinations among communities of color.

As language is another barrier to COVID-19 vaccination, it is important to provide information through trusted messengers within the community, develop cultural appropriate materials, and make the materials available in multiple languages [76]. Other recommendations to make vaccines more accessible include to require language access for the most commonly spoken languages in a district, provide professional translations for information on COVID-19 websites, ensure informational websites are available in different languages, and to offer language translation assistance at vaccination sites [75]. In addition, incorporating providers that serve sizable numbers of immigrant families as vaccine administration sites may promote access and lessen possible language barriers among participants [76].

### 5.3. Bridge the Digital Divide

Not having access to an adequate internet connection could result in people who attempt to schedule a vaccination appointment online experiencing longer than intended loading screens and the need to refresh the link to the appointment site repeatedly [85]. Therefore, people may consider convenience factors and ponder how easy it is to make a vaccination appointment and sign in [94]. This is likely to be time-consuming and frustrating. The time consumption and frustration could lead to those individuals not taking part in vaccination. Therefore, it is important to simplify registration procedures and provide vaccination options that do not require preregistration to ensure equity in the distribution of vaccines [85]. 

Vaccination appointments at sites in most affected communities should also be prioritized for residents in that community. There should be consideration for the fact that ease of virtual signup may attract people from wealthier neighborhoods who have more reliable internet access and fewer access barriers, and efforts made to ensure that lack of access to a computer or the internet does not pose a barrier to vulnerable communities [79,87]. To address digital divide, administrators should also offer alternative registration methods for COVD-19 vaccination, including government assurance of offline access to registration appointments and enlistment of community organizations, healthcare workers, or clergy to help register people in person or over the phone for vaccine appointments [95].

### 5.4. Partner with Trusted Local Sources

Other strategies to address the impacts of structural racism and promote vaccine awareness and uptake within marginalized communities include partnering with trusted local sources such as community organizations, local health care institutions, and faith-based organizations [85,96]. To facilitate vaccine knowledge and uptake within local communities, special consideration should be paid to organizations and institutions that serve historically marginalized communities who have shouldered a disproportionate burden of COVID-19 exposure, morbidity, and mortality [85]. There should be a concerted attempt to leverage trusted community leaders to engage Black and Hispanic communities in public health campaigns in order to decrease mistrust.

Educating the community must effectively utilize the multiple ways people are open to considering the messages. Research has shown that many religious leaders are trusted sources of information among Black populations [97]. In addition to traditional sources of information, Black Americans are two to three times more likely than Whites to trust charitable organizations and religious leaders [98]. Older Black Americans report considerably more trust than Whites in informal information sources for health care information, including family or friends and church or religious leaders [99]. There is also a need to train local, multilingual community health workers to fill the gaps in community-based COVID-19 response efforts and educate and connect people to health care services [100]. Receiving health messages from trusted sources may influence motivation and, ultimately, the decision to get vaccinated [94].

Furthermore, while it is useful to include trusted voices as part of marketing campaigns, genuine collaboration requires a commitment to fully engaging and integrating community leaders and organizations into the planning and implementation of solutions. True partnership involves partner organizations working side by side with health care specialists and public health collaborators to promote use of preventive services [18]. When organizations engage affected community members, residents can feel empowered rather than discouraged and disenfranchised. Confronting inequities in vaccine uptake necessitates a multifaceted approach focused on the needs of traditionally marginalized communities and structures of racism that breed that mistrust and influence vaccine rollout. Strategies must acknowledge that vaccine contemplation is embedded in structural racism and must be devised to clearly illustrate the trustworthiness of health care systems, medicine, and public health [18]. 

### 5.5. Provide Convenient and Trusted Points of Acccess 

Prioritizing the most affected groups and increasing vaccination clinic availability is needed to improve vaccination rates in Black and Hispanic populations. There is a continued need for federal, state, and local efforts to make vaccines accessible and to provide education and outreach about vaccines to address racial and ethnic gaps COVID-19 vaccination [12]. It is important to ensure that vaccination sites are easily accessible, in multiple formats (e.g., walk-up, drive-up), and during a variety of hours, including evenings and weekends [76]. It is also important to meet people where they are, and this can include utilizing old-fashioned methods, such as neighbors talking to neighbors, flyers, and pastors speaking with church members [78]. Convenient and trusted points of access to the COVID-19 vaccine can include places where community members commonly gather, such as schools, community centers, faith-based organizations, or mobile vaccination units organized by trustworthy and credible community-based organizations in publicly-accessible spaces, such as a park or town hall [79]. There should also be provision of workplace COVID-19 vaccination programs, which can prevent COVID-19 illness, reduce absenteeism and doctor visits due to illness, and improve employee morale [101]. 

## 6. Conclusions

The disproportionate impact of COVID-19 on Black and Hispanic communities commands a multifaceted investigation into how structural racism affects access to and confidence in the COVID-19 vaccine. In this paper, we detail the detrimental effects of structural racism on health and racial and ethnic disparities in health. Further, we detail how the COVID-19 pandemic has revealed the unequal health effects of structural racism among Black, Hispanic/Latino, and American Indian/Alaska Native American populations. Additionally, we detail common structural barriers to COVID-19 vaccine access and how they disproportionately affect Black and Hispanic populations. We also propose recommendations related to educational, policy, clinical, and community-engaged approaches to reduce racial and ethnic disparities in COVID-19 vaccine uptake. Overall, confronting disparities in vaccine uptake requires a multifaceted approach focused on the needs of historically marginalized communities. Strategies must acknowledge that vaccine access is embedded in structural racism and must be devised to clearly exhibit the credibility of health care systems, medicine, and public health [18].

## Figures and Tables

**Table 1:** Common structural barriers to COVID-19 vaccination.

Barrier	Description/Explanation
Convenience	Lack of a centralized system for residents to register for and schedule vaccination appointments [43] Complexity of vaccine scheduling system related to internet access, comfort with technology, distance to travel, and accommodations for older adults and people with disabilities [43]
Language	Language requirements and burdensome proof-of-eligibility requirements increase difficulties for some immigrants [41] Varying translation help at vaccination clinics [75] Inaccurate translation on health department websites [75]
Immigrant status	Non-citizen immigrants are more likely to be concerned about potential costs related to obtaining the vaccine compared to U.S. citizens [76] Potential barriers to vaccination include lack of flexibility in work and childcare responsibilities, limited transportation options, lack of access to computers, and literacy and language challenges [63,76] Fear that is caused by collecting information from immigrants at COVID-19 vaccination sites [42]
Transportation/Difficulty traveling to a vaccination site	Vaccination sites are not always near public transit and not everyone has access to a car [41] Ride-sharing can be costly [41] Lack of accommodation for homebound individuals, older adults, and people with disabilities [43]
Computer/Internet access	Poor internet access makes securing a vaccination appointment a challenge [43] Access to a traditional computer and home broadband differs by race and ethnicity [77] Environmental services staff (e.g., janitors) may not have access to hospital email [78]
Lack of accessible and trusted points of access (clinic location)	Lack of prioritization of disproportionately affected groups has led to limited vaccination clinic availability in Black and Hispanic neighborhoods [16] Initial phases of vaccine distribution have been primarily limited to chain pharmacies and large health-care systems, which are significantly less prevalent in Black communities [79]

## Data Availability

No new data were created or analyzed in this study. Data sharing is not applicable to this article.
